# How Institutional Evaluation Bridges Uncertainty and Happiness: A Study of Young Chinese People

**DOI:** 10.3389/fpsyg.2021.651844

**Published:** 2021-10-13

**Authors:** Ying Wu, Guangqiang Qin, Chuanyi He, Wenying Wang

**Affiliations:** ^1^School of Ethnology and Sociology, Minzu University of China, Beijing, China; ^2^Department of Psychology, Faculty of Arts and Science, Queen's University, Kingston, ON, Canada

**Keywords:** institutional evaluation, uncertainty, happiness, age difference, young people

## Abstract

Uncertainty triggers negative psychological responses, while positive institutional evaluations elevate the sense of control in individuals and satisfy their need for structure and order. Data from the 2015 Chinese Social Survey (CSS) (*N* = 4,605) demonstrated that objective uncertainty negatively predicted the happiness of young people (aged 18–45 years). However, this negative relationship was attenuated among those who evaluated the institutional system (e.g., social security, local government effectiveness, and trust in government) positively; in other words, positive institutional evaluation may have protected people's happiness from the threat of uncertainty. In addition, participants from different age groups evaluated the institutional system differently. The first generation born after the Chinese economic reform, which includes young people born in the 1980s (aged 26–35 years), had unique experiences compared to the preceding (aged 36–45 years, born in the 1970s) and succeeding (aged 18–25 years, born in the 1990s) generations. Among the three age groups, young people born in the 1980s held the least positive evaluation of the institutional system. The institutional evaluation also showed the weakest moderating effect on this group's happiness.

## Introduction

The COVID-19 pandemic has increased the sense of uncertainty among people across the world, resulting in greater intergroup conflicts and extreme behaviors. Several studies have reported that people experienced more severe psychological distress and reduced well-being and increased alcohol use during the pandemic (Kikuchi et al., 2020; Tran et al., 2020; De Sio et al., 2021). The cumulative effects of uncertainties result in negative, or even extreme, psychological responses among people. Studies have shown that objective uncertainty factors, such as economic depression, war, epidemics, unemployment, interpersonal stress, and other adverse life events, can trigger pessimism and weaken positive psychological responses (Ballas and Dorling, 2007; Proctor et al., 2009; Hogg et al., 2013; Bianchi, 2016).

On the other hand, social systems allow individuals to experience higher levels of satisfaction and happiness by increasing positive emotions in people (Wakslak et al., 2007). Internalizing the legitimacy of social systems could elevate the sense of control in individuals and satisfy their need for structure and order (Jost and Banaji, 1994). A given social system comprises various social arrangements, such as those constituting families, institutions, organizations, governments, and nature. These arrangements include both institutions and social norms, such as *de facto* institutional structures, systems of allocation, and socialization, as well as governments, judicial systems, and other substantive forms of social existence (Jost and Banaji, 1994). When uncertainties in life lead to perceptions of randomness and fluctuating perceptions of personal control, the social system as a robust external order could reduce the randomness (Kay et al., 2008). According to the Human Development Report (United Nations Development Programme, 2014), active governmental response to the needs of citizens, comprehensive public policies, and instructive social norms are beneficial to reducing psychological vulnerabilities, feelings of injustice, and other negative emotions and exclusionary behaviors. A positive institutional evaluation may buffer the negative effects of uncertainty in life and enable people to gain positive psychology.

Young people face a number of life changes, such as graduation, employment, marriage, fertility, and other issues, which make them susceptible to significant uncertainty. In China, due to the rapid social development and social transformation, as well as the process of marketization, large-scale population migration, mobility, and the popularization of Internet technology, young people have experienced greater uncertainty from changes in their social environment. Studies from China have found that there are differences among young Chinese people based on their values, social participation, trust, democratic consciousness, and sense of fairness (Li, 2019). For young people of China, could institutional evaluation relieve the adverse effects of uncertainty? Does the buffering effect of institutional evaluation differ among young people of different age groups?

### From Uncertainty to Happiness

Research has suggested that the perception of uncertainty can trigger negative psychology. In particular, uncertainty over identity can elicit extreme behaviors (Hogg and Adelman, 2013; Hogg et al., 2013) and feelings of unfairness and doubt in the justice system (van den Bos, 2001). Uncertainty also fosters religious fanaticism and extremist ideologies (McGregor et al., 2010, 2013). Objective uncertainty (e.g., as a result of bereavement, the end of a relationship, or unemployment) can compromise happiness and life satisfaction (Ballas and Dorling, 2007; Ruthig et al., 2007; Luhmann et al., 2012). Furthermore, uncertainty promotes a shift in the beliefs of individuals from individualism towards collectivism, causing them to seek support from external groups and collectives (Bianchi, 2016).

Most research on uncertainty and happiness has focused on subjective uncertainty (van den Bos, 2001; Hogg, 2007; Doosje et al., 2013), with less attention paid to objective uncertainty. It is possible that subjective uncertainty is more significant than objective uncertainty, as the former may have a greater impact on the responses of individuals. However, a relationship between objective uncertainty and happiness could be more powerful considering a less individualized measure of uncertainty and its broader practical implications. For example, it could remind policy-makers to modify existing systems to tackle objective uncertainty. In the present study, we examine, among other factors, unemployment, real estate purchases, illness, and parental caretaking responsibilities as our measures of objective uncertainty with the aim of bridging the gap in extant literature.

### Institutional Evaluation as a Bridge Between Uncertainty and Happiness

Economics and business management research have examined various objective indicators of government effectiveness and their effects on happiness, with higher levels of governmental efficiency, impartiality, and quality, as well as lower levels of institutional corruption, found to be positively associated with improved happiness among the general population (Helliwell and Huang, 2008; Bjørnskov et al., 2010; Altindag and Xu, 2011; Kim and Kim, 2011). In addition, governmental spending on social expenditures such as public health, educational systems, and social welfare systems have been found to be effective in promoting social security, as well as happiness among people (Hessami, 2010; Kotakorpi and Laamanen, 2010). Research has also focused on the trust of individuals in social institutions to examine the effects of institutional evaluation on happiness. Portela et al. (2013) found that social trust and institutional trust are positively related to people's happiness. Using a Ghanaian sample, Sulemana (2014) found that institutional trust can promote happiness. Furthermore, Hudson (2006) pointed out that a higher level of institutional trust is beneficial in promoting a stable, optimistic outlook and elevating happiness.

As highlighted in compensatory control theory (CCT), uncertainty and chaos in the environment tend to trigger an intense need for structure and order among individuals (Kay et al., 2008, 2009). According to CCT, benevolent governments, as part of the external social system, are capable of compensating for the low sense of control in individuals (Kay et al., 2008). Governmental agencies and systems can serve as robust and stable sources of external order, motivating individuals to reestablish their sense of control. In turn, these external systems can satisfy the need for structure and order among individuals. Individuals with a lower sense of control are likely to be more reliant on external social systems and defend the legitimacy of such systems (Jost and Banaji, 1994). Napier and Jost (2008) found that political conservatives reported higher levels of happiness compared to liberals; furthermore, this relationship was moderated by a greater tendency among conservatives to justify existing social inequalities.

Despite being based on CCT, the current research differs from previous research in the field. We focus on the impact of uncertainty on the positive psychological reactions of people and whether external social systems could alleviate the impact of uncertainty on the psychology of people. Our study uses survey data to verify whether evaluations of institutional systems by people can buffer the negative effects of uncertainty, whereas most previous studies have used the experimental paradigm to explore how virtual uncertainty could motivate people to follow external social systems (Kay et al., 2008). We hypothesize that positive institutional evaluation will act as affirmative cognition to satisfy the need for structure and order in individuals, attenuating the negative psychological effects of uncertainty, and thus elevating their overall level of happiness.

### Buffering Effect of Institution Evaluation on Age Differences

Studies on happiness have found that, in Western samples, age has a significant impact on happiness (Mroczek and Kolarz, 1998; Stone et al., 2010). This effect is U-shaped, where the young and elderly demographics display elevated levels of happiness, while the middle-aged adults display the lowest levels of happiness.

Similarly, in the context of uncertainty, differences have been noted among age groups in terms of evaluation of the government by individuals. A survey conducted during the COVID-19 pandemic showed that, around the globe, the youngest cohort (16–24 years of age) was the most supportive of government policies related to COVID-19, while those in the most economically active group (25–39 years of age) were least supportive of government policies. People in the age group of 40–65 years, with mostly positive attitudes, were categorized between the other two groups (Chamier et al., 2020).

In China, there are obvious generational differences that indicate “social generations” constructed by social contexts. Studies have highlighted differences in the beliefs and values of those born and raised after the reform and opening-up, in the post-1970s, post-1980s, and post-1990s, from those born and raised post-1950s and post-1960s. The former group constitutes China's New Generation, who were simultaneously influenced by and played a critical role in a series of historical events that occurred in the aftermath of the reform and opening-up (Fan, 2012; Li, 2019). Since we used data from 2015, the corresponding age groups of those born post-1970s, post-1980s, and post-1990s were 36–45 years, 26–35 years, and 18–25 years, respectively.

The group of young people (18–45 years) has a span of around 30 years, and there would be differences within them due to age or generation. The group of young people born in the 1980s (26–35 years) is unique in being the first generation born after China's reform and opening-up. From the perspective of age, young people born in the 1980s are in a stage of life where problems related to employment, marriage, and childbirth are concentrated, and so the sense of control and stability expectations given by the institution may be weaker. The buffering effect of the institution may be weaker in reducing uncertainty and increasing happiness in young people born in the 1980s when compared to the two young groups born in the 1970s and 1990s, respectively. We hypothesize that the moderating effect of institution evaluation of post-1980s is weaker than that of post-1970s and post-1990s.

### Hypotheses

We examined the association between uncertainty and happiness in young Chinese people. Based on a sample from the 2015 CSS, we tested the following hypotheses:

H1: Objective uncertainty is negatively associated with happiness.
H2: Higher levels of institutional evaluation are positively associated with happiness.
H3: The relationship between uncertainty and happiness is moderated by institution evaluation, that is, positive institution evaluation reduces the negative impact of uncertainty on happiness.
H4: The moderating effect of institution evaluation of post-1980s is weaker than that of post-1970s and post-1990s.

## Methods

### Data and Sample

The current data set was obtained from the open database of the 2015 Chinese Social Survey (CSS). The CSS is an annual, large-scale continuous sampling survey project that was launched by the Institute of Sociology, Chinese Academy of Social Sciences, in 2005. The purpose of the CSS was to conduct a long-term longitudinal survey on the employment, family, and social life of the people, and social attitudes across 31 provinces/cities/autonomous regions, in both urban and rural areas, covering a total of 604 villages/communities and 151 counties/districts across China. A total of 10,268 individuals completed the survey. As the research considers only young adults, we focus our analyses on respondents aged 18–45 years (*N* = 4,605).

### Variables

#### Dependent Variable

##### Happiness

One item in the CSS measured happiness: “Overall, I am a happy person.” Responses ranged from “Strongly disagree” (1) to “strongly agree” (5), with higher scores corresponding to higher levels of happiness (M_happiness_ = 3.64, SD = 0.96).

#### Independent Variables

##### Objective Uncertainty

One question in the CSS measured objective uncertainty (see Table 1): “Which of the following issues have you or your family experienced in the past 12 months?” Ten items were listed, with each describing an uncertain circumstance. An item was coded “1” if it was selected by the respondent and “0” if it was not. Following standard practice, we added the scores of the respondents from all 10 items, yielding a 10-point measure for objective uncertainty. Higher scores represented higher levels of objective uncertainty. In the current sample, the mean uncertainty score was 2.69 (SD = 2.11).

**Table 1:** Objective uncertainty measure.

Which of the following issues have you or your family experienced in the past 12 months? (Multiple answers can be selected)
Poor housing conditions; cannot afford to build/purchase housing
Educational cost for children is high and difficult to maintain
Poor familial relations (i.e., divorce, separation, conflicts between mothers, and daughters-in-law)
Medical costs are high and difficult to maintain
Price inflation affects standards of living
Low household income, causing difficulties in daily living
Family members are unemployed, fired or working in unstable jobs
Burdened by expenses related to supporting elderly family members
Interpersonal expenses are high and difficult to maintain
Encountering fraud, theft or other crimes

##### Institutional Evaluation

Institutional evaluation of individuals was measured based on five questions in the CSS (see Table 2). Since institutional evaluation consists of various indicators, such as the degree of trust in relevant governing bodies and evaluations of social welfare systems and local government efficiency (α = 0.78), we drew upon item response theory (IRT) to reveal the latent traits of individuals in institutional evaluation from the above indicators and placed individuals on a measurement scale of 0 to 10. IRT is a widely used model which assumes that respondents have an unobserved trait that is expressed through their responses to a series of questions. The value of this index ranges from 0 to 10, with a higher value indicating a more positive institutional evaluation. Indices constructed using the IRT are more accurate than those constructed using the traditional summation method (Ayala, 2008).

**Table 2:** Institutional evaluation measure.

Individuals' evaluation of the institutional system was computed based on responses to the following items: trust in relevant governing bodies; evaluations of social welfare systems and local government efficiency.
To what extent do you find police officials trustworthy? (rated between “extremely untrustworthy” and “extremely trustworthy” on a five-point scale)
To what extent do you find judges trustworthy? (rated between “extremely untrustworthy” and “extremely trustworthy” on a five-point scale)
To what extent do you find government officials trustworthy? (rated between “extremely untrustworthy” and “extremely trustworthy” on a five-point scale)
Overall, how would you evaluate the current social welfare system? (rated between “extremely dissatisfactory” and “extremely satisfactory” on a five-point scale)
Overall, how would you rate the efficiency of your local government? (rated between “extremely dissatisfactory” and “extremely satisfactory” on a five-point scale)

#### Control Variables

We considered the following as control variables: gender (female = 1), age (18–25 years = 0; 25–35 years = 1; 35–45 years = 2), ethnicity (Han ethnicity = 1; other ethnic minority = 0), years of education (M = 10.35; SD = 4.04), marital status (married = 1; other status = 0), *Hukou* (household area: urban = 1; rural = 0), and employment status (employed = 1; other status = 0).

Among the various demographic variables, age is most likely to be the defining indicator of in-group diversity in our cohort of young people. In the statistical analyses of existing literature, two approaches—considering age as a continuous variable (age) and a categorical variable (age group)—have been used. Grouping people by age is a common statistical strategy, especially in sociological literature (Nan and Heath, 1995; Matthijs and Kalmijn, 2017). In this article, we regard age as a grouping variable, with the aim of examining heterogeneity among young people based on the analysis of the overall situation of the demographic. If age is used as a continuous variable, only one-dimensional coefficients can be obtained, which would not allow the internal heterogeneity of young people to be assessed in detail. In addition, our sample size is large enough (*N* = 4,605) to support our analysis of heterogeneity among younger groups.

Based on the Chinese context, at the age of 25 years people usually complete their master's education and begin their career. Cohorts aged 25–45 years may exhibit certain in-group differences since within this time period individuals often transition from holding entry-level jobs to having an established career; similarly, individuals often transition from seeking relationship partners to having a stable family life. Hence, we divided the sample population into three age groups: 18–25 years (young people born in the 1990s), 26–35 years (young people born in the 1980s), and 36–45 years (young people born in the 1970s). Subsequently, we examined the moderating and optimizing effects of institutional evaluation on the relationship between objective uncertainty and happiness, separately for each age group. Prior to our analysis, we compared demographic factors across the age groups (Table 3) and found statistically significant differences in employment and marital status, whereas the three stable demographic factors (i.e., gender, ethnicity, and *Hukou* status) were evenly distributed. The results further justify our current method of splitting the sample based on the age of the respondents.

**Table 3:** Demographic distribution of present sample across age groups.

	**Employment status** **(employed)**	**Marital status** **(married)**	**Gender** **(female)**	**Ethnicity** **(Han)**	***Hukou*** **(urban)**	**Education** ***M*****(***SD***)**
18–25 (*n* = 901)	40.7%	26.1%	48.1%	90.2%	32.6%	12.44 (3.26)
26–35 (*n* = 1478)	72.1%	85.1%	41.7%	91%	33.3%	10.89 (3.94)
36–45 (*n* = 2226)	80.7%	92.9%	43.4%	91.2%	34.3%	9.12 (3.96)
χ^2^	492.76^***^	1708.77^***^	9.28^**^	0.73	0.89	*F* = 264.06^***^

*** *p < 0.001*,

** *p < 0.01*.

The complete list of descriptive statistics is shown in Table 4. In the present sample, 48.1% of the respondents were female, 33.64% were registered under an urban *Hukou*, and 77.33% were married. The proportion of ethnic minorities was small, as the respondents were predominately Han Chinese. The average number of years of education was 10.35 (SD = 4.04), and 70.12% of the respondents were employed.

**Table 4:** Descriptive statistics of all variables (*N* = 4,605).

**Variable**		**Mean/Percentage**	***SD***
Gender	female = 1; male = 0	49.21%	
Age	18–25 = 0	19.57%	
	26–35 = 1	32.10%	
	36–45 = 2	48.34%	
*Hukou*	Urban = 1	33.64%	
	Rural = 0	66.36%	
Marriage	Married = 1	77.33%	
	Others = 0	22.67%	
Ethnicity	Han people = 1	91.00%	
	Ethnic minority = 0	9.00%	
Education	0–19 years	10.35 years	4.04
Employment	Employed = 1	70.12%	
	Others = 0	29.88%	
Happiness (1–5)	Percentage (Happiness = 4 + Happiness = 5)	61.11%	
Uncertainty (0–10)		2.69	2.11
Institutional evaluation (1–10)		5.14	1.84

### Statistical Models

Since the dependent variable, happiness, in the present study is presented as a five-level ordinal variable, we adopted ordinal logistic regression in our multivariate statistical analysis. We began by building a baseline model before adding other variables of interest, moving from the lowest to the highest level. Specifically, the baseline model (Model 1) consisted only of control variables; two crucial explanatory variables—objective uncertainty and institutional evaluation—were then added to Model 2. We further expanded Model 2 to examine the moderating effects of institutional evaluation on the relationship between uncertainty and happiness, and how these moderating effects differ significantly across all three age groups. In Model 3, we incorporated a two-way interaction between uncertainty and happiness, as well as a three-way interaction among the age group, objective uncertainty, and institutional evaluation. Notably, the continuous variables in the models (objective uncertainty, institutional evaluation, and years of formal education) were found to be zero-centered.

## Results

### Relationship Between Varying Levels of Institutional Evaluation, Exposure to Objective Uncertainty, and Happiness Among Young People

Logistic regression parameters are generally estimated using the maximum likelihood method. Likelihood values reflect a given model's goodness-of-fit, where a higher goodness-of-fit reflects a greater model fit. In view of this, we compared the relative goodness-of-fit of our three nested models by performing a log-likelihood chi-square test. On comparison, Model 2 and Model 1 yielded a likelihood ratio of 310.96 (*df* = 2, *p* = 0.000), suggesting that Model 2 was a significantly better fit than Model 1; Model 3 and Model 2 yielded a likelihood ratio of 8.40 (*df* = 3, *p* = 0.045), suggesting that Model 3 was a significantly better fit than Model 2. The latter also suggested that the overall fit of the model was boosted by incorporating two-way and three-way interactions and thus contained better explanatory power than did models that only examined the main effects.

As shown in Table 5, Model 1 was a baseline model that primarily examined the associations between demographic variables and happiness. The results also showed that happiness was lower among males when compared to females; happiness decreased with age and increased with years of education. Married people in the sample showed a higher level of happiness. Ethnicity, *Hukou*, and employment status were not statistically significant in the sample, indicating that the level of subjective happiness was not significantly different across these factors.

**Table 5:** Ordinal logistic model for happiness.

	**(1)** **Control variables only**	**(2)** **Control variables + main effect**	**(3)** **Control variables + moderating effects**
	m1	m2	m3
Gender (female = 1)	0.184^**^	0.158^**^	0.157^**^
	(3.20)	(2.75)	(2.72)
Age (26–35)	−0.620^***^	−0.505^***^	−0.522^***^
	(6.45)	(5.22)	(5.38)
Age (36–45)	−0.706^***^	−0.615^***^	−0.623^***^
	(7.07)	(6.14)	(6.20)
Han Ethnicity (minority = 0)	0.088	0.053	0.057
	(0.90)	(0.54)	(0.57)
Education	0.067^***^	0.056^***^	0.056^***^
	(7.89)	(6.53)	(6.55)
Married (other = 0)	0.611^***^	0.632^***^	0.631^***^
	(7.08)	(7.30)	(7.29)
Hukou (rural = 1)	0.004	−0.050	−0.053
	(0.07)	(0.75)	(0.80)
Employment	0.029	−0.071	−0.070
	(0.45)	(1.09)	(1.08)
Uncertainty		−0.209^***^	−0.207^***^
		(14.88)	(14.41)
IE		0.104^***^	0.104^***^
		(6.66)	(6.60)
Uncertain × IE			0.043^*^
			(2.41)
Age (26–35) × uncertain × IE			−0.050^*^
			(−2.37)
Age (36–45)*uncertain*IE			−0.029
			(−1.43)
Cut1	−3.428^***^	−3.609^***^	−3.629^***^
	(24.29)	(25.12)	(25.22)
Cut2	−2.041^***^	−2.176^***^	−2.190^***^
	(16.63)	(17.40)	(17.48)
Cut3	−0.384^**^	−0.440^***^	−0.449^***^
	(3.25)	(3.67)	(3.74)
Cut4	1.709^***^	1.736^***^	1.727^***^
	(14.19)	(14.19)	(14.11)
N	4605	4605	4605
Log likelihood	−6029.962	−5874.484	−5870.465
LR chi2	179.23	490.19	498.22
Prob > chi2	0.000	0.000	0.000
Pseudo R2	0.040	0.041	0.064

*t statistics in parentheses*.

*^+^p < 0.1*,

* *p < 0.05*,

** *p < 0.01*,

*** *p < 0.001; IE, Institutional Evaluation*.

The associations between objective uncertainty and institutional evaluation and levels of subjective happiness were examined in Model 2, after controlling for gender, age, marital status, *Hukou*, and employment status. The results revealed that the level of objective uncertainty was negatively correlated with happiness, whereas institutional evaluation was positively correlated with happiness. For every one-point increase in objective uncertainty, while controlling for all other variables, the odds ratio of increasing happiness by one additional level decreased by 18.9% (e^−0.209^-1); for every one-point increase in institutional evaluation, the odds ratio of increasing happiness by one additional level increased by 11.0% (e^0.104^-1).

Model 3 was the full model. In this model, a two-way interaction (between uncertainty and institutional evaluation), as well as a three-way interaction (between age, uncertainty, and institutional evaluation), were introduced after controlling for demographic variables. The results once again revealed that uncertainty and institutional evaluation were significantly correlated with happiness. Notably, the interaction coefficient for the two-way interaction was 0.043, which was significant, with *p* < 0.05. This suggests that the association between objective uncertainty and happiness was significantly moderated by institutional evaluation.

**Figure 1:**
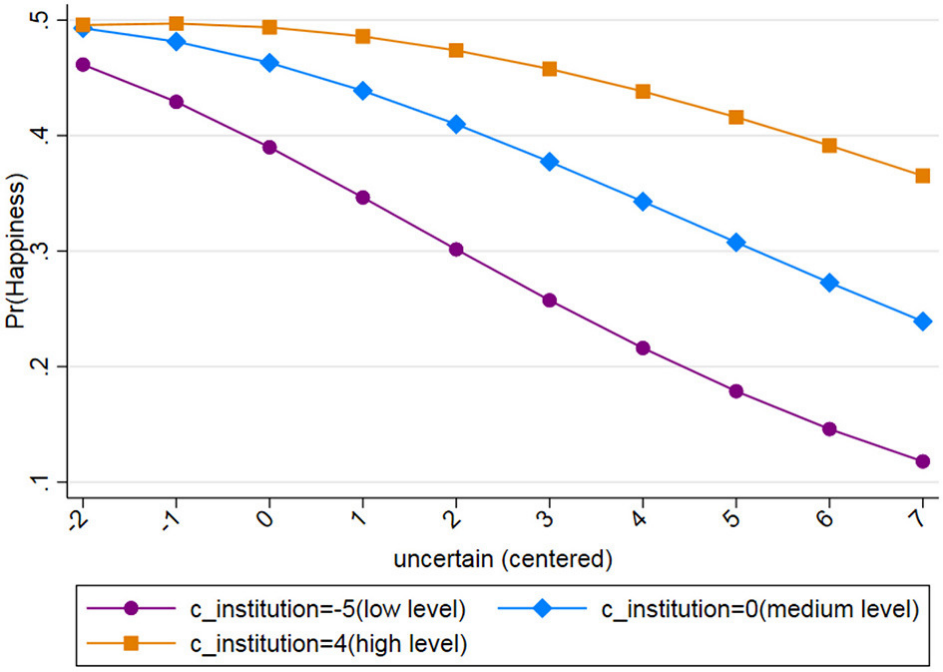
The relationship between uncertainty and happiness under different levels of institutional evaluation.

**Figure 2:**
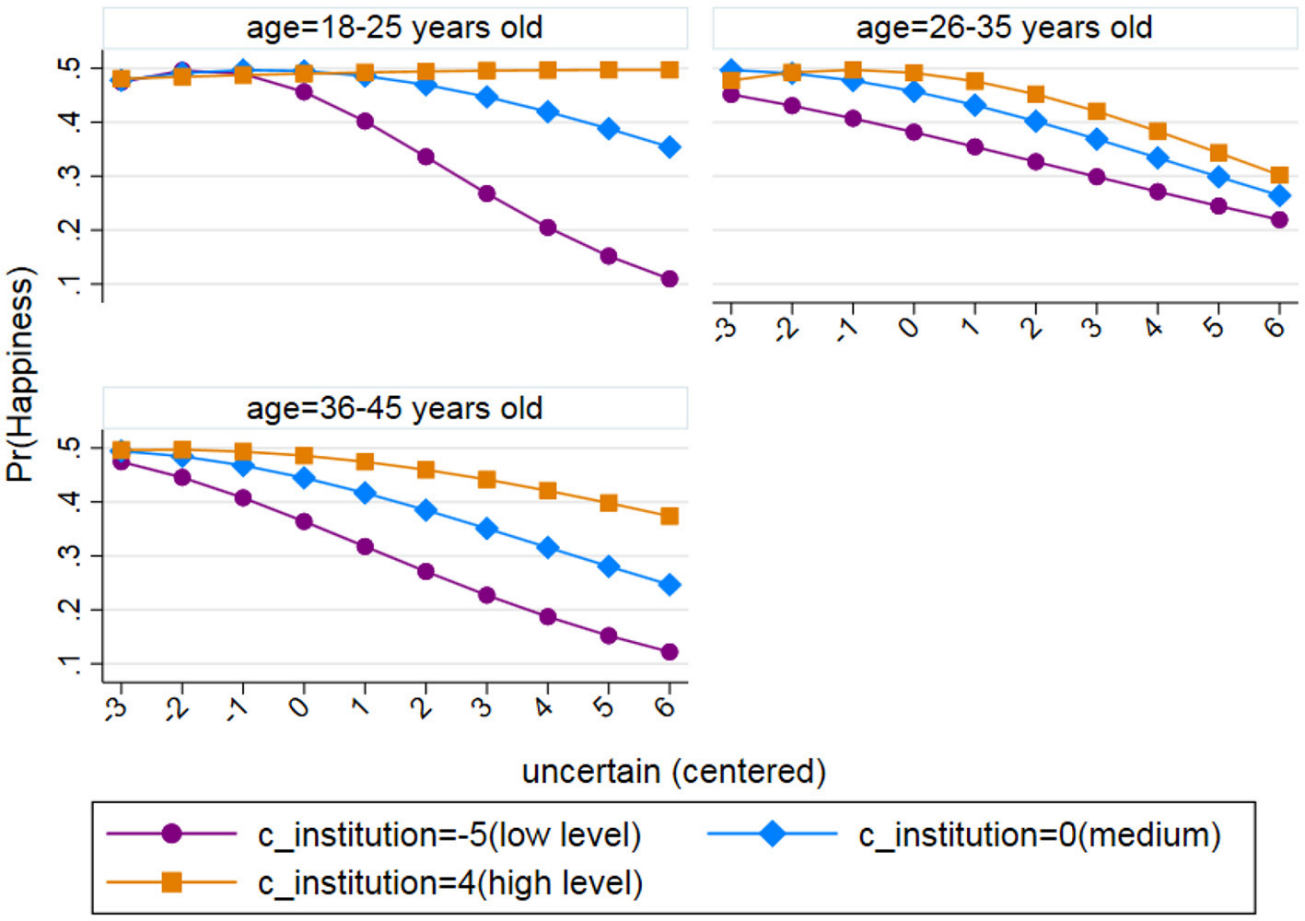
Differences in the moderating effect of institutional evaluation for three age groups.

With an increase in institutional evaluation scores, the extent to which life uncertainty predicted happiness changed accordingly. We provide a more detailed analysis in the following section by plotting the moderating effect (see Figure 1). Additionally, drawing on interaction coefficients from the three-way interaction, we show that the cohort aged 26–35 years had an interaction coefficient of −0.05 (*p* < 0.05), while the interaction coefficient for the cohort aged 36–45 years was not statistically significant. The results suggest that the moderating effect of institutional evaluation on uncertainty and happiness was significantly different between age groups. The effect was significantly different between the cohort aged 26–35 years and the cohort aged 18–25 years, while it was not significantly different between the cohort aged 36–45 years and the cohort aged 18–25 years. Figure 2 provides a visual representation of these results.

### Analysis of the Moderating Effects of Institutional Evaluation

In Model 3, we conducted an analysis of the moderating effect of institutional evaluation on uncertainty and happiness. After the data were centralized, the variable range for institutional evaluation in our analysis was between −5 and 4. To better illustrate the moderating effect of a specific variable, we selected three data points within the range of this continuous variable: (1) data point 4, which represented the highest evaluation of the institutional system; (2) data point 0, which represented the average level of institutional evaluation; and (3) data point −5, which represented the lowest evaluation of the institutional system. Each of the three curves in Figure 1, from top to bottom, represents the relationship between uncertainty and happiness at each of the three data points. All three curves exhibit a gradual negative trend, indicating that the levels of happiness decrease with an increase in the levels of objective uncertainty. Under different levels of institutional evaluation, however, there are significant differences in the steepness of the slope decline. When the institutional evaluation of individuals is relatively positive, the slope of the curve is at its flattest (β = −0.05); in other words, the negative association between uncertainty and happiness is at its most minimal. In contrast, when the level of institutional evaluation is relatively low (β = −0.392), the slope of the curve is the steepest. When the level of institutional evaluation is at the intermediate level (β = −0.202), the negative association between uncertainty and happiness also lies between the two extremes. Thus, the plot indicates that high levels of institutional evaluation could significantly lower the negative relationship between uncertainty and happiness.

### Differences in Moderating Effect in Different Age Cohorts

The present study compared the respondents' scores for happiness, objective uncertainty, and institutional evaluation across three age groups. The results showed that with increasing age there was also a significant increase in the exposure of respondents to objective uncertainty. Specifically, those in the age group of 36–45 years showed significantly higher exposure compared to those in the age group of 18–25 years (*F* = 21.72, *p* < 0.00). In terms of institutional evaluation scores, there was also a significant difference between age groups (*F* = 9.87, *p* < 0.00). However, the overall trend was different. As shown in Table 6, those in the age group of 18–25 years displayed the highest level of institutional evaluation, while those in the age group of 26–35 years displayed the lowest level of institutional evaluation and those in the 36–45 years group displayed an intermediate level of institutional evaluation.

**Table 6:** Comparison of variable scores of different age groups.

	**Happiness**	**Uncertainty**	**IE**
	**%**	***M*** **(***SD***)**	***M*** **(***SD***)**
18–25 (*n* = 901)	67.93	2.66 (2.15)	5.30 (1.74)
26–35 (*n* = 1478)	61.31	3.11 (2.25)	4.98 (1.81)
36–45 (*n* = 2226)	58.41	3.24 (2.26)	5.18 (1.88)
*F*		21.72^***^	9.87^***^

*** *p < 0.001*,

^**^p < 0.01; IE, Institutional Evaluation.

### Analysis of the Three-Way Interaction Between Age, Institutional Evaluation, and Objective Uncertainty

The relationship between institutional evaluation and age was non-linear since the moderating effect of institutional evaluation on uncertainty and happiness differed across age groups. A plot representing the potential three-way interaction is illustrated in Model 3. We presented the moderating effect of institutional evaluation on uncertainty and happiness in three groups that were split according to the age of the respondents, which included cohorts of 18–25-year-olds, 26–35-year-olds, and 36–45-year-olds, respectively. In examining the interaction coefficients, the moderating effect of the institutional evaluation was significantly different between the cohort of 26–35-year-olds and the cohort of 18–25-year-olds (see Table 4). The moderating effect was stronger in the cohort of 18–25-year-olds, which is reflected in Figure 2. In the cohort of 18–25-year-olds, specifically, the top curve is almost horizontal to the x-axis, suggesting that when individuals rated the institutional system more positively, the negative association between uncertainty and happiness was weaker, and when individuals rated the institutional system less positively, the negative association between uncertainty and happiness was more pronounced (the third curve on the plot for the cohort of 18–25-year-olds shows a steep negative slope). In other words, the moderating effect of institutional evaluation on uncertainty and happiness was most salient in the cohort of 18–25-year-olds. From a policy-making perspective, increasing institutional evaluation in young people aged 18–25 years yielded the highest rate of return in terms of increasing subjective happiness.

Conversely, the three curves are most closely clustered for the cohort of 26–35-year-olds, suggesting that the moderating effect of institutional evaluation on uncertainty and happiness was the weakest for this cohort. Additionally, the three-way interaction coefficients showed that there was no significant difference between the cohort of 36–45-year-olds and the cohort of 18–25-year-olds in terms of moderating effect (see Table 4). Thus, the moderation of institutional evaluation on uncertainty and happiness was also effective for the cohort of 36–45-year-olds. The three curves show clear divergence; the negative association between uncertainty and happiness was noticeably weaker under high institutional evaluation in comparison to low institutional evaluation. Taken together, this shows that the moderating effect of the high institutional evaluation was more pronounced for the cohorts of 18–25- and 36–45-year-olds. Consequently, improving their institutional evaluation would help them achieve higher levels of happiness.

## Discussion

On analyzing a representative sample of Chinese young adults, we found that greater objective uncertainty was associated with reduced happiness and positive institutional evaluation was associated with increased happiness, consistent with past research using Western samples (Hudson, 2006; Portela et al., 2013; Sulemana, 2014). In addition, the present study revealed that institutional evaluation moderated the negative association between objective uncertainty and happiness among young people in China, i.e., positive institutional evaluation predicted a weaker negative association between objective uncertainty and happiness.

The results also echo previous findings from system justification theory (Jost and Banaji, 1994; Jost et al., 2004; Napier and Jost, 2008) and CCT (Kay et al., 2008, 2009, 2010) on social systems. Our results suggest that the institutional system, as part of the external social system, is capable of providing individuals with psychological support and a buffer, so that their need for structure and order can be met in the face of high uncertainty. Simultaneously, institutional evaluation can weaken the negative association between uncertainty and happiness.

The current research differs from previous CCT studies in several ways. First, CCT only proposed that uncertainty increases people's dependence on external systems but did not discuss the influence of external systems on people's psychological results in uncertain contexts. Our study used empirical research to further verify that the system can weaken the negative results of uncertainty and therefore promote happiness in people. Second, unlike the existing experimental paradigm, we used large-scale survey data to test the effect of the promotion of external systems on the positive psychology of people. Third, most external systems mentioned in previous studies have been abstract (Kay et al., 2008). Conversely, our study collated Chinese survey data based on trust in the system, performance evaluation of local government, and evaluation of social welfare, which was beneficial in making people understand what kind of external social system in real society could reduce the negative impact of uncertainty.

The results of the present study are consistent with previous findings on uncertainty, namely, objective uncertainty elicits negative psychological states in individuals (van den Bos, 2001; Hogg, 2007; Doosje et al., 2013). By introducing institutional evaluation as a variable in uncertainty research, and by examining its mitigating effects on negative psychological outcomes, the present study proposed another way for individuals to maintain happiness amid objective uncertainties.

The present study is of practical significance for several reasons. First, according to our results, high institutional evaluation can buffer the negative results caused by uncertainty, and positive institutional evaluation can boost people's happiness. This finding can be applied in the current context of the COVID-19 pandemic; through institutional construction, people's trust and evaluation of the system can be improved and the negative psychological consequences caused by the pandemic can be mitigated. Previous studies have also found that people's dependence on and expectations of external systems have increased during the pandemic. For example, the uncertainty caused by the pandemic led to increased support of Donald Trump, the President of the United States (Jones, 2020), as well as enhanced trust in the formal social system (Kye and Hwang, 2020).

Second, during the process of China's modernization and social transformation, people have been paying greater attention to formal institutional construction. Positive institutional effectiveness has become a pillar for the Chinese to deal with uncertainty, as it helps to alleviate negative emotions in uncertain situations and enhance people's happiness. Therefore, institutional construction, including the effect of local governance, social security systems, and institutional trust, plays an important role in promoting positive social mentality in people and maintaining social harmony and stability.

Third, the buffering effect of institutional evaluation on uncertainty is different for every age cohort. On the one hand, it may be related to the generational differences in the Chinese context. China's rapid social change entails differences in values, social participation, and institutional evaluation among people of different generations. As a special group and social generation, young people have their own characteristics. Their lives have been marked by major social changes, such as the one-child policy, rapid economic growth, the rise of the Internet, education expansion, marketization, urbanization, and globalization, which have had a considerable impact on their living circumstances and opportunities, shaping their generational characteristics while widening the intergenerational gap between them and previous generations (Li, 2019).

Moreover, our study suggested that the positive moderating effect of the institutional evaluation was weakest among the generation born in the 1980s. Young people born in the 1980s had to face immense pressures from marketization, including the pressure of employment brought about by the expansion of China's higher education system; half of the members of this population were employed in non-state-owned enterprises and a considerable proportion was in a state of floating employment. This generation also had to deal with high real estate prices (Fan, 2012). These factors resulted in a weakening of the impact of institutional evaluation among young people born in the 1980s when compared to preceding and succeeding generations.

According to the results of our research, the young people born in the 1980s (26–35-year-olds) scored lower institutional evaluation and higher uncertainty. The buffering effect of lower institutional evaluation on higher uncertainty was not too strong. Moreover, the weaker buffering effect could be due to the age stage or generation experience of the people born in the 1980s. From the perspective of the age cohort, they are at a stage where employment, marriage, and childbirth issues arise simultaneously; while 18–25-year-olds are yet to enter the stage completely, and most of the people aged 36–45 years have completed the major life tasks. From the perspective of generation difference, the institutional evaluation of young people born in the 1980s was weaker. So it means that the stable expectation and sense of control brought by the institutional evaluation would be less.

The findings of our study have practical and policy implications, and this means by giving the young people more stable institutional expectations and better institutional guarantee matching their life stages, which could help them to reduce the uncertainty and contribute to social stability. Based on the results of the present research, relevant policies need to be introduced in employment, housing, children's education, and social security to meet the needs of different age groups involved in the construction of China's future institutions, as well as the diversified needs of young groups, allowing greater opportunities for social participation and public practice decision-making.

On the other hand, each age group among the young people has different life development tasks, which may also result in different buffering effects of institution evaluation. The different effects of institution evaluation among the young group of people were also applicable beyond the Chinese context. The group of 26–35-year-olds is economically active and faces pressures related to the market and economy, which may be the reason for their lower institutional evaluation and the weaker effect of institutional evaluation. A recent survey on institutional trust amid the COVID-19 pandemic showed that respondents aged 26–39 years were the least satisfied with the government's behavior, which may explain the commonality of institutional evaluation in this age group around the world (Chamier et al., 2020).

For young people with high uncertainty and low institutional evaluation, corresponding psychological interventions could be carried out to raise their positive mentality. Firstly, enhancing the sense of control among young people, training the young people's growth thinking and positive attribution cognitive model, as well as a high sense of self-efficacy, could relieve the negative psychological results caused by uncertainty. Secondly, at the group level, cultivating the national identity of young people, and providing opportunities for social participation to improve their institutional evaluation, could buffer the negative results brought about by uncertainty in life.

The study is also subject to several limitations. First, it is based on an existing survey, and thus we did not have control over the measures. Second, the research is correlational, meaning that we were unable to draw causal conclusions. Future research may examine the causal relationships among institutional evaluation, uncertainty, and happiness. Finally, samples from other cultures may be included to test the generalization of the present findings.

## Conclusion

In conclusion, data from Chinese young adults suggest that positive institutional evaluation can buffer the negative association between uncertainty and happiness among individuals. We also found that respondents of different age groups have significantly different institutional evaluations and that age influences the moderating effect of institutional evaluation in respondents.

## Data Availability Statement

Publicly available datasets were analyzed in this study. This data can be found here: http://css.cssn.cn/css_sy/zlysj/lnsj/201706/t20170615_3551582.html Chinese Social Survery.

## Author Contributions

YW formulated the research question. GQ, YW, and WW analyzed the data. YW and CH wrote the manuscript, with input from all coauthors. All author read and approved the final manuscript.

## Conflict of Interest

The authors declare that the research was conducted in the absence of any commercial or financial relationships that could be construed as a potential conflict of interest.

## Publisher's Note

All claims expressed in this article are solely those of the authors and do not necessarily represent those of their affiliated organizations, or those of the publisher, the editors and the reviewers. Any product that may be evaluated in this article, or claim that may be made by its manufacturer, is not guaranteed or endorsed by the publisher.
